# Beyond Left and Right: Binding and Retrieval of Spatial and Temporal Features of Planned Actions

**DOI:** 10.5334/joc.197

**Published:** 2022-01-06

**Authors:** Viola Mocke, Patricia Holzmann, Bernhard Hommel, Wilfried Kunde

**Affiliations:** 1 Department of Psychology, University of Würzburg, Germany; 2 Department of Psychology, University of Leiden, Netherlands

**Keywords:** action, action control, feature binding, action planning, preparation

## Abstract

There is evidence that planning an action relies on binding codes of the relevant features of that action into an action plan. Such binding is indicated by the observation that planning a novel action is impaired if it shares some but not all features with another action that is held in memory for later execution. Most previous studies have focused on the binding of the spatial features *left* and *right*, which are particularly salient but not the only features of intentional body movements. In a series of four online experiments, we tested whether evidence for the binding of other (non)spatial features could also be found. Taken together, we indeed obtained evidence for the binding of temporal (*short* and *long*) and vertical (*top* and *bottom*) features, in addition to the commonly studied horizontal features (*left* and *right*). Yet, clear binding effects were mainly restricted to action features that remained uncertain up to the point the respective action had to be planned. These observations have important theoretical and methodical implications for the future studies of feature binding in action planning.

Planning an action means to set up an internal representation of the to-be-executed action that allows the agent to swiftly carry it out whenever necessary. These representations are commonly called action plans. Action planning has been argued to consist in binding action features into action-specific event files or action files (Hommel et al. 2001). The creation of action files is often studied in situations in which the agent plans an Action A for later execution, but first initiates another more or less similar Action B before carrying out the planned Action A. If planning Action A comprises of bindings of the codes of its features, executing Action B should be hampered if its features partly overlap the prepared Action A—as binding the feature codes into a plan is assumed to “occupy” these codes and thus interfere with using the same codes for other purposes (Stoet & Hommel 1999). Hence, feature overlap between Actions A and B should produce so-called partial overlap costs (Frings et al. 2020).

An early series of experiments by Stoet and Hommel (1999) demonstrates this phenomenon most clearly. Participants planned a particular Action A for later execution with one hand (releasing the home key, that is, the central one of three keys, with one hand, pressing another of the three keys, and returning to the home key), carried out another Action B with the same or the other hand (releasing and pressing the home key), and finally carried out the planned Action A. Despite absence of any motor activity related to Action A, reaction time for B was slowed down if A was planned to be performed with the same hand. This suggests that binding the code of the feature *left* or *right* into action plan A in some sense occupied this code until the execution of A, so that using the same code for the planning of Action B was impaired. In a second experiment, Action A was again carried out with the left or right hand while Action B consisted of a left or right pedal response. Again, Action B was more slowly initiated if it shared the location feature with the planned Action A. What these experiments suggest is that planning actions defined by their horizontal location involves the binding of corresponding horizontal feature codes into action plans.

## (Non-)Spatial Action Features

While partial overlap costs have been demonstrated in numerous experiments, almost all of them studied the overlap of the spatial features *left* and *right* (e.g., Fournier, Behmer & Stubblefield 2014; Fournier, Wiediger & Taddese 2015; Mattson & Fournier 2008; Mattson, Fournier & Behmer 2012; Stoet & Hommel 1999; Wiediger & Fournier 2008; see Fournier et al. 2010 for verbal responses). Representing motor actions in spatial terms appears natural, as carrying out an action often means to move a limb in space. Especially the spatial terms *left* and *right* are used from childhood on to tell apart homologous limbs of our body (e.g., *left* and *right* hand). Thus, horizontal features appear particularly apt to represent motor actions and become bound to other action-related features. Yet, spatial features need not be horizontal, and there are ways to describe actions other than by their spatial characteristics, such as in terms of temporal features. Thus, the question remains whether empirical evidence for the binding of features other than the horizontal features *left* or *right* can be demonstrated.

Whereas there is evidence that motor actions that vary in temporal terms, such as short and long keypresses, can be prepared in advance (Klapp & Jagacinski 2011; Kunde 2003), it is less clear whether the corresponding codes of temporal features are bound into action plans. On the one hand, temporal *stimulus* features have been shown to be bound to *left* or *right* response features (Bogon, Thomaschke & Dreisbach 2017). On the other hand, however, there are observations suggesting that temporal features are not that easily bound into event files. Specifically, congruency-sequence effects, which result from partial feature overlap between previous and current stimulus-response (S-R) events do occur in spatial (Hommel, Proctor & Vu 2004) but not in temporal interference tasks in which responses are *short* or *long* (Kunde & Stöcker 2002). As outlined by Bogon and colleagues (2017), duration is an inherently dynamic feature. For example, while a stimulus (or action) can be qualified as *left* or *right* from the onset of that stimulus (or action), it can be described as *short* or *long* only after some time. Assuming that action plans describe intended perceptual feedback of a motor pattern (Stoet & Hommel 1999), it might take somewhat longer, at least as long as the coded short duration, to represent and bind the features *short* and *long* into an action plan. Another aspect that renders temporal features interesting to study is that action durations can be construed as being continuous on top of being categorical. That is, *short* and *long* keypresses both vary within a certain defined interval. While this continuous nature might be true for spatial features like *left* and *right* as well, in the typical experimental setups, these spatial features describe categorical choices (i.e., fixed response locations). Whether feature codes refer to categorical response alternatives or to responding within an acceptable range of values might thus also be a source of differences in binding and/or retrieval effects related to spatial and temporal features. Considering the described differences between spatial and temporal features and the fact that previous findings of partial overlap costs related to *left* and *right* features, we were particularly interested in checking whether partial feature overlap costs differ between spatial and temporal features. As especially spatial features seem to be natural and potentially indispensable parts of action plans (i.e., plans to move a limb in space) and because of previous research suggesting that temporal features are less likely integrated in event files (Kunde & Stöcker 2002), we hypothesized stronger binding and retrieval, reflected in larger partial overlap costs, for spatial (even vertical) than temporal features. Besides looking at feature overlap costs for each of these feature types, differences between these types would be most clearly expressed by an interaction of feature overlap (none vs. partial) and feature type (vertical vs. temporal).

Taken together, the role of binding spatial features other than *left* or *right* and temporal features is still unclear. We addressed this question by investigating whether, and to which extent, partial-overlap costs arise for vertical spatial features or temporal features, and whether they differ.

## Experiment Overview

We carried out four online experiments in total. All data and analyses are available on the *Open Science Framework* (*https://osf.io/7qyh4*; Mocke et al. 2021) and all experiments were preregistered (Experiment 1: *https://osf.io/xjuq8*; Experiment 2: *https://osf.io/tf6zy*; Experiment 3: *https://osf.io/zcrbu*; Experiment 4: *https://osf.io/8kue6*). We report how we determined our sample size, all data exclusions (if any), all manipulations, and all measures in the studies. All experiments were performed in accordance with the Declaration of Helsinki (Rickham 1964) and had been approved by the local ethics committee (Ethikkommission des Institutes für Psychologie der Humanwissenschaftlichen Fakultät der Julius-Maximilians-Universität Würzburg, GZEK 2019-39). All participants gave their informed consent before participation.

**Figure 1:**
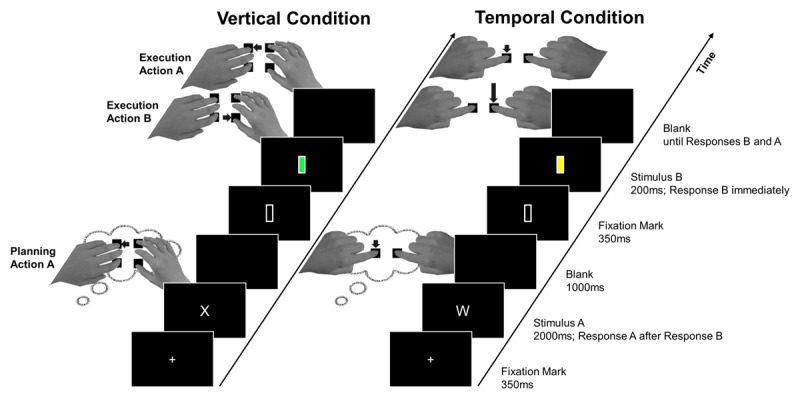
Example Trial for Each Block Applicable to All Four Experiments. *Note*: Upon presentation of Stimulus A (here: ‘X’ in the spatial and ‘W’ in the temporal condition), the corresponding Action A (see below) was to be planned (for later execution). As soon as Stimulus B (here: green square in the spatial and yellow square in the temporal condition) appeared, the corresponding Action B was to be carried out, followed immediately by execution of the planned Action A. The figure shows a no-overlap trial. The stimulus-response mapping was as follows. *Vertical condition*. Participants responded using their index and ring fingers of one hand and their thumb and middle finger of the other hand. In Experiments 1 to 3, a letter stimulus indicated a *left*-hand response, ‘X’ with the *top* (R key) and ‘O’ with the *bottom* (C key) key. Color stimuli indicated a *right*-hand response with ‘red’ signaling a *top* (U key) and ‘green’ a *bottom* (N key) keypress. In Experiment 4, a letter stimulus indicated a *top* keypress, ‘X’ a *left*-hand response (R key) and ‘O’ a *right*-hand response (U key). Color stimuli indicate a *bottom* keypress with ‘red’ signaling a *left* (C key) and ‘green’ a *right* (N key) keypress. *Temporal condition*. Participants responded using their index fingers. In Experiments 1 to 3, a letter stimulus indicated a *left*-hand response (D key), ‘W’ a *short* and ‘U’ a *long* keypress. Color stimuli indicated a *right*-hand response (J key) with ‘blue’ signaling a *short* and ‘yellow’ a *long* keypress. In Experiment 4, a letter stimulus indicated a *short* keypress, ‘W’ a *left*-hand response (D key) and ‘U’ a *right*-hand response (J key). Color stimuli indicated a *long* keypress with ‘blue’ signaling a *left* (D key) and ‘green’ a *right* (J key) keypress.

In all four experiments, there was a *vertical* and a *temporal* condition (see ***Figure 1***). Both conditions comprised a different response set of four possible finger actions. Each action was uniquely defined by the combination of two features. One of these features referred to the action’s horizontal (*left* or *right*) location, which was true for both the vertical and the temporal condition in all four experiments. The other of the two features either referred to the action’s vertical (*top* or *bottom*) location, in the *vertical* condition, or its duration (*short* or *long*), in the *temporal* condition. In short, in each experiment, in the *vertical* condition, the four possible actions were defined by combinations of their horizontal and vertical location, and in the *temporal* condition, the four possible actions were defined in terms of their horizontal location and their duration.

**Figure 2:**
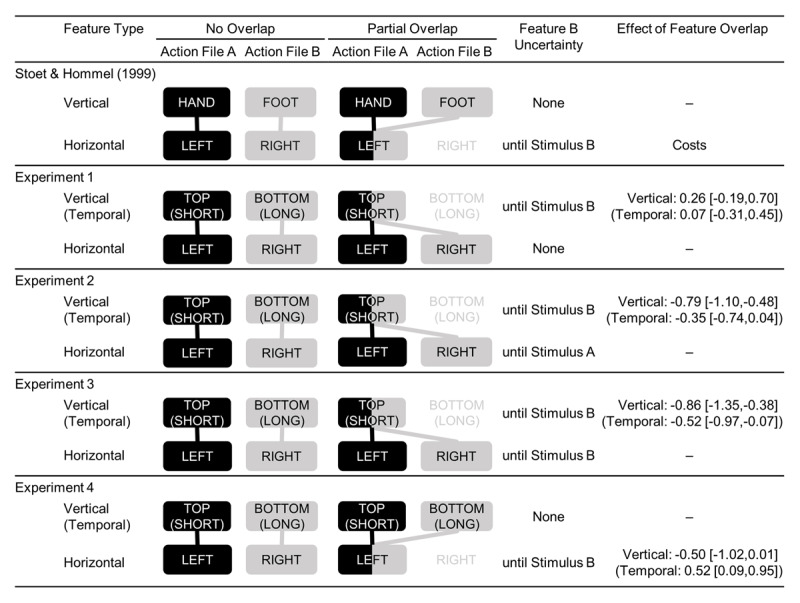
Action Features Studied by Stoet and Hommel (1999, Experiment 2) and the Present Four Experiments. *Note*: Both conditions in each experiment comprised a horizontal (*left*/*right*) feature. Conditions differed regarding the second feature type, that is, either vertical (*top*/*bottom*) or temporal (*short*/*long*). The illustrations show examples of no-overlap versus partial-overlap trials. Lines depict proposed feature bindings. The right columns denote the degree of uncertainty about features needed for the first required Action B, and whether partial overlap effects in the vertical (temporal) condition were observed. Negative values mean costs, positive values mean benefits of feature overlap. Confidence intervals of the paired differences are given in square brackets (Pfister & Janczyk 2013).

Participants were asked to prepare (but not yet carry out) Action A upon presentation of Stimulus A, to initiate Action B upon presentation of Stimulus B, and then to immediately carry out the planned Action A. The two actions did or did not overlap in terms of one (or, in Experiment 3, even both) of their features. The experiments mainly differed regarding the uncertainty of the features identifying each action, or, more precisely, whether features of Action B were uncertain, and if so, up to which point in a trial that uncertainty remained. ***Figure 2*** summarizes these differences and displays exemplary feature combinations in the partial- and no-overlap conditions for each experiment, including the reference study by Stoet and Hommel (1999, Experiment 2).

### General Method

#### Participants

Fluent German speakers from the general German population were recruited via Prolific Academics. Participants performed the task online on their individual Desktop devices. Participation was financially compensated with GBP6.75. Throughout the experimental series, we excluded those datasets that showed clear signs of low participant engagement, such as no or only one correct trial in one or both experimental blocks and datasets of participants who after finishing the experiment reported having used different fingers in the spatial block than demanded by the instructions.

The effect of interest in the current experiments is the 2 × 2 interaction between feature overlap and feature type, that is, the difference between overlap costs in the vertical and the temporal condition. To our knowledge, previous literature has not made this specific comparison yet, so we used an effect size estimate from the reaction time (RT) data of a similar pilot experiment (*d_z_* = 0.44, *n* = 40), which had yielded partial-overlap benefits in the temporal condition and no partial-overlap costs in the spatial condition. To be on the safe side and be able to detect smaller effect sizes as well, we set the maximum sample size to *n* = 70 (corresponding to *d_z_* = 0.34, with a power of 1 – β = .80 and an alpha level of α = .05, calculated with G*Power, Faul et al. 2007) and decided to terminate data collection if the data provided strong support for either the H_1_ or the H_0_ (i.e., BF_10_ > = 10 or < = .1, Jeffreys 1961). For this purpose, one-sided Bayesian paired *t*-tests for the described comparison were calculated in JASP 0.10.2 (JASP Team 2019) every ten participants. The results of these tests for all experiments and measures are summarized in the online material (*https://osf.io/7qyh4*; Mocke et al., 2021). The prior was conservatively described by a Cauchy distribution centered around zero and with a default width parameter of .71, which corresponds to a probability of 80% that the effect size lies between 0 and 2.

#### Apparatus and Stimuli

In the vertical condition, participants responded using their ring and index finger of one hand and their middle finger and thumb of the other hand (see ***Figure 1***). Specifically, participants placed their thumb and index finger on the C and N keys and their middle and ring fingers on the R and U keys on a QWERTY-based keyboard. The use of non-homologous fingers within a vertical feature category (*top*–*bottom*) served the purpose of eliminating the confounding factor of finger homology (Stinear, Walker & Byblow 2001). If they had used homologous fingers, then in the overlap condition after planning action A, they would use the homologous finger of the other hand for action B, while in the no overlap condition, a non-homologous finger would be used. This could potentially result in a data pattern appearing like overlap costs or benefits. The assignment of which fingers were used on which hand was counterbalanced across participants. In the temporal condition, participants placed their left and right index fingers on the D and J keys. Here, the use of (non-)homologous fingers would apply to both conditions of no and partial temporal feature overlap, thus not being a confounding factor. We used homologous fingers to simplify instructions.

Each response was defined by the combination of one horizontal feature (*left* or *right*) and either a vertical feature (*top* or *bottom*)—in the vertical block—or a temporal feature (*short* or *long*)—in the temporal block. While horizontal and vertical features were inherently defined by the position of the effector finger/keys they referred to, we defined short responses as between 0 and 200 ms and long ones as between 201 and 500 ms.

We used two different stimulus types (i.e., letters and colors). In Experiments 1 to 3, the two stimulus types were, counterbalanced across participants, assigned to the two horizontal (*left* and *right*) features, and in Experiment 4 to the two vertical (*top* and *bottom*) or the two temporal (*short* and *long*) features. Within each stimulus type, the specific stimulus then indicated the other required feature (*vertical* or *temporal* in Experiment 1 to 3, and *horizontal* in Experiment 4), randomly across participants. Letter stimuli were, in the spatial (temporal) block, ‘X’ and ‘O’ (‘W’ and ‘U’) in white Arial font taking 10% of the respective monitor’s height. Color stimuli were, in the spatial (temporal) block upright red or green (yellow or blue) rectangles with a white outline sized 3.75% × 10%. Letter stimuli were always preceded by a white fixation cross (3% × 3%) and color stimuli by the white rectangle outline of the color stimulus.

#### Procedure

Each experiment contained a blocked vertical and temporal condition with order of conditions counterbalanced across participants (***Figure 1***). Both conditions followed three training blocks (for letter stimuli only, color stimuli only and the final task) that were not included in the analyses. Both conditions consisted of four mini-blocks, after which participants could take short breaks. Each trial started with the respective fixation symbol depending on the stimulus type A. After 350 ms, Stimulus A appeared and stayed on screen for 2000 ms followed by a 1000 ms blank. During that time, participants could plan the respective Action A. Then, the fixation symbol for Stimulus B showed for another 350 ms followed by Stimulus B which was on screen for 200 ms. With its onset, participants could respond to this second stimulus with Action B, immediately followed by the previously planned Action A. This sequence (press B, release B, press A, release A) had to be carried out within 5000 ms after onset of Stimulus B. After an intertrial interval of 1250 ms, the next trial started automatically.

In case of an error, a brief error message appeared (1000 ms) and the trial terminated immediately. Trials were considered erroneous when the participant responded prematurely (i.e., before the onset of Stimulus B), or failed to finish the sequence of keypresses within time. This could happen because participants failed to execute Action A or Action B at all (omission error) or gave the incorrect response (commission error, i.e., the response key in the spatial block or the keypress duration in the temporal block were incorrect). In addition, participants could respond with the wrong hand or, but only in the temporal block, press the correct response key longer than 500 ms. Error frequencies and outliers are publicly available online (*https://osf.io/7qyh4*; Mocke et al. 2021).

#### Design and Data Analysis

The experiments followed a 2 × 2 within-subjects design with the independent variables feature overlap (none vs. partial), manipulated trial-wise, and feature type (vertical vs. temporal), varied block-wise. Initially, we planned to keep the dependent variables as similar as possible to the ones by Stoet and Hommel (1999), that is, RTs, error rates (ERs) and keypress durations as dependent measures for Action B and Action A. After having conducted Experiment 2, we decided post-hoc to partly deviate from this preregistered strategy for the following reasons.

**Figure 3:**
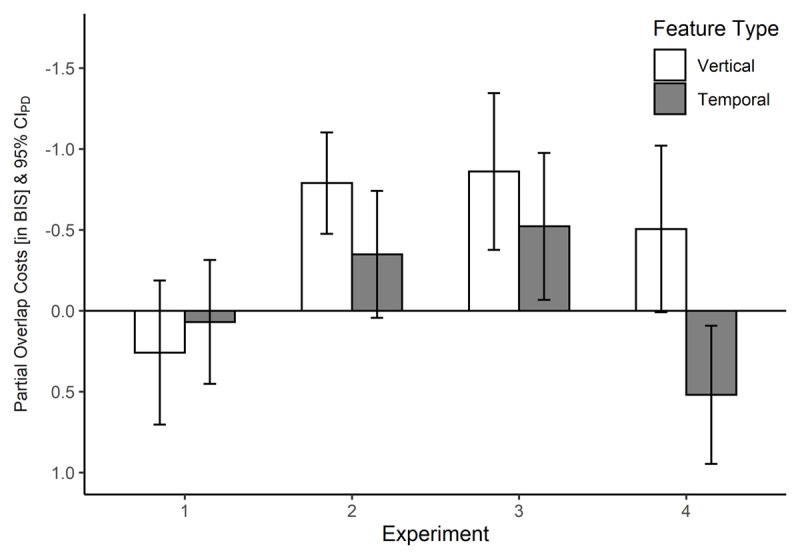
Partial Overlap Costs (Partial-Overlap – No-Overlap) Across the Four Experiments. *Note*. Negative values denote partial overlap costs, positive values denote benefits (Note the reversed y-axis). In the relevant trials of Experiments 1 to 3, Actions A and B could partly overlap with respect to vertical or temporal but not to horizontal features. These experiments differ regarding the uncertainty of the horizontal Feature B. In Experiment 4, action files could partly overlap with respect to horizontal features but not to (the certain) vertical or temporal features. Error bars represent confidence intervals of the paired differences (see Pfister & Janczyk 2013).

Firstly, in foreshadowing of the results, RTs and ERs showed rather different effect patterns, possibly suggesting various kinds of trade-offs. Overlap effects were sometimes more strongly expressed in RTs and sometimes more in ERs—without a recognizable pattern. We thus decided to use Balanced Integration Scores (BIS), which cancel out speed-accuracy tradeoff effects while retaining real effects (Liesefeld & Janczyk 2019). It is this measure we will focus on in the following, but we provide the descriptors for all three measures (RTs, ERs, and BIS) for all experiments in ***Table 1***. For the calculation of BIS, RT and ER data were preprocessed in the following, preregistered manner. Trials in which participants did not execute the correct sequence of responses were not included in individual participants’ response time cell means. Of all correct trials, those in which response times deviated 2.5 standard deviations (SDs) or more from the participant’s respective 2 (feature type) × 2 (Feature A) × 2 (Feature B) response time cell mean for the respective action were excluded from all analyses. For comparability with the results by Stoet and Hommel (1999), ERs were computed as all trials with incorrect response divided by the number of trials, in which participants saw the respective stimulus. BIS were then calculated as {z_{PC}} - {z_{\overline {RT} }} with percentage correct (PC) being 1 – ER. Standardised values were based on SDs calculated per subject over all 2 × 2 conditions (in line with Vandierendonck 2021). The here reported analyses are 2 × 2 repeated-measures analyses of variance (rmANOVAs) for Action B BIS. In case of a significant interaction, we will report the results of follow-up *t*-tests for both feature types separately. ***Figure 3*** shows the obtained partial-overlap effects across conditions and experiments. Results of the same rmANOVAs for RTs and ERs individually can be found online (*https://osf.io/7qyh4*; Mocke et al., 2021).

**Table 1:** Means (Standard Deviations) of All Dependent Measures by Condition and Experiment.


DEPENDENT MEASURE	VERTICAL CONDITION		TEMPORAL CONDITION	

	OVERLAP	NO OVERLAP	OVERLAP	NO OVERLAP

Experiment 1 (*n* = 50)				

Hand Alternation				

BIS	–0.49 (1.27)	–0.75 (1.29)	0.65 (1.06)	0.58 (1.35)

RT	658 (136)	678 (149)	597 (131)	609 (127)

ER	5.9 (4.4)	5.3 (4.8)	4.7 (5.1)	4.7 (5.2)

Experiment 2 (*n* = 70)				

Hand Alternation				

BIS	–0.82 (1.24)	–0.03 (1.13)	0.25 (1.29)	0.60 (1.28)

RT	771 (261)	755 (274)	704 (212)	709 (231)

ER	12.6 (8.9)	10.8 (8.4)	8.9 (5.8)	7.4 (6.3)

Experiment 3 (*n* = 50)				

Hand Alternation				

BIS	–0.69 (1.16)	0.17 (1.33)	0.00 (1.30)	0.52 (1.12)

RT	876 (265)	820 (233)	848 (349)	844 (320)

ER	11.8 (8.5)	10.7 (8.9)	10.7 (11.1)	7.2 (8.6)

Hand Repetition				

RT	636 (116)	785 (230)	697 (182)	830 (277)

ER	1.4 (2.6)	10.7 (11.9)	3.5 (5.1)	8.3 (7.3)

Experiment 4 (*n* = 40)				

Hand Alternation				

BIS	–0.19 (1.16)	0.31 (1.13)	0.20 (1.12)	–0.32 (0.97)

RT	602 (119)	599 (122)	591 (126)	590 (139)

ER	7.2 (7.7)	5.5 (5.0)	6.6 (7.3)	9.3 (7.8)


*Note*: Mean values with standard deviations in parentheses. Balanced integration scores (BIS, Liesefeld & Janczyk 2019) are the combination of reaction times (RTs) and error rates (ERs) in hand alternation trials per feature overlap condition. In Experiments 1, 2 and 4, there were hand alternation trials only. To increase feature uncertainty regarding the features *left* and *right* there were also hand repetition trials in Experiment 3. Yet, for the sake of comparability with the other experiments, only hand alternations trials were analyzed in detail. The higher the BIS, the better the performance.

Secondly, even though our preregistration included an rmANOVA of movement durations as reported by Stoet and Hommel (1999), we decided to refrain from presenting it here. As keypress durations are a harder to interpret indicator of performance than the movement durations in the original study, we decided to make these results openly accessible online and instead report an additional exploratory analysis. This applies to the temporal condition in the first three experiments only and yields insights into how prepared Action A plans including an abstract duration feature impact keypress duration in Action B. Specifically, we conducted a 2 (Feature A) × 2 (Feature B) rmANOVA for keypress durations, to shed light on whether the duration of an action, being continuous, is torn towards or away from the duration category represented in a prepared action plan. We are not aware of research studying how the inclusion of a temporal feature in an action plan impacts the execution of other actions that use either the same or a different temporal feature. Basically, attraction and repulsion effects appear tenable. Attraction would mean that planning a short keypress results in overall shorter duration of another concurrent action. Repulsion means the opposite, that planning a short keypress might result in overall longer duration of another concurrent action.

Thirdly, for the sake of brevity, we will not report results of all preregistered and above-mentioned additional analyses conducted for Action A instead of B. These can be found online (*https://osf.io/7qyh4*; Mocke et al. 2021).

## Experiment 1

We conducted the first experiment (a) to replicate the partial overlap costs found by Stoet and Hommel (1999) in a spatial condition using overlapping vertical (*top* or *bottom*) instead of horizontal (*left* or *right*) features, and (b) to compare these costs with those in a temporal condition with overlapping temporal (*short* or *long*) features. Action A always required a *left* action, while Action B always required a *right* action, or vice versa (see ***Figure 2***). Accordingly, horizontal feature overlap was always zero and there was always complete certainty regarding the association between the horizontal features and the Actions A and B, respectively. Uncertain was which of the actions were associated with the feature *top* or *bottom* (in the vertical condition) or the feature *short* or *long* (in the temporal condition). Accordingly, Actions A and B could overlap with respect to vertical (or temporal) features, and whether there was any overlap became apparent not before presentation of Stimulus B. Based on previous experiments (e.g., Stoet & Hommel 1999), we expected worse performance in partial-overlap than in no-overlap trials, and we were interested to see whether partial-overlap costs would be comparable across vertical and temporal conditions.

### Method

#### Participants

Data collection was stopped after having a final sample of *n* = 50 participants (14 female, 34 male, 2 other, M_age_ = 28.2, range = [18,64]), as the RT data provided already strong support for the H_0_, BF_01_ = 12.89, Median Cohen’s *d* = 0.05, 95% credibility interval (CI) = [0.00, 0.22]. From the collected *n* = 61 complete data sets, as preregistered, we had excluded participants who showed more than 30% errors in the temporal block (*n* = 4), in the vertical block (*n* = 6), or in both blocks (*n* = 1).

#### Procedure

In each trial, participants first saw a letter stimulus (e.g., ‘X’ in the spatial or ‘W’ in the temporal condition) and planned the respective Action A (e.g., *top* in the spatial or *short* in the temporal condition) with the according (e.g., *left*) hand. Subsequently, based on a color stimulus (e.g., green in the spatial or yellow in the temporal condition), they planned and initiated Action B (e.g., *bottom* in the spatial or *long* in the temporal condition) with the other (e.g., *right*) hand (see ***Figure 1***).

Note that, in this first experiment, Action A stimuli were always letters and Action B stimuli always colors. As each stimulus type was mapped to one horizontal feature and because a color stimulus always followed a letter stimulus, the hand sequence in each trial was fixed (*left – right* or *right* – *left*). Both the temporal and the spatial conditions consisted of four mini-blocks each containing 40 trials, hence comprising 80 partial-overlap and 80 no-overlap trials each.

### Results

#### Balanced Integration Scores

The 2 (feature overlap) × 2 (feature type) rmANOVA for BIS showed overall worse performance in the vertical than the temporal condition, *F*(1,49) = 18.59, *p* < .001, \eta _p^2 = .28. Importantly, neither the main effect of overlap, *F*(1,49) = 1.33, *p* = .26, \eta _p^2 = .03, nor its interaction with feature type were significant, *F*(1,49) = 0.40, *p* = .53, \eta _p^2 = .01.

#### Response Durations

The exploratory 2 (Feature A) × 2 (Feature B) rmANOVA of Action B durations in the temporal condition showed that keypresses B were longer when Action B required a *long* compared to a *short* keypress, *F*(1,49) = 1333.63, *p* < .001, \eta _p^2 = .97. This merely shows that participants followed the instructions. Yet, there was also a repulsion effect of the temporal feature of the planned Action A, with longer durations of Action B when a *short* rather than *long* Action A was planned, *F*(1,49) = 13.24, *p* = .001, \eta _p^2 = .21. The latter effect was stronger for *long* keypresses (332 ms with a *short* and 324 ms with a *long* keypress planned) than for *short* ones (119 ms with a *short* and 118 ms with a *long* keypress planned), *F*(1,49) = 8.62, *p* = .005, \eta _p^2 = .15.

### Discussion

The results of Experiment 1 were unexpected. Despite employing a very similar paradigm as in previous studies on action planning (e.g., Stoet & Hommel 1999), there were no consistent partial-overlap costs, neither for vertical nor temporal features. Of course, it is possible that neither of these features, as opposed to horizontal features, become integrated in action plans in a detectable manner. Yet, on reflection of the employed paradigm we considered another possibility.

Feature binding should be particularly relevant when there is uncertainty about which features must go together at any given point in time. Yet, in the present design the horizontal hand feature (*left* or *right*) for both Action A and B was certain already before the stimulus onset (in fact, already when reading the instructions). Throughout the entire experiment, the hand sequence was fixed (*left – right* or *right* – *left*). Such complete certainty about the horizontal feature for any action file in the entire experiment might have discouraged binding and/or retrieval of those features, or changed the planning process so to construct one coherent plan for both responses. Note though, that the same level of feature certainty applied to the features *hand* and *foot* in the original study by Stoet and Hommel (1999, Exp. 2), posing a question that we will answer in Experiment 4. Still, with this idea in mind, we tried to reduce feature certainty in a second experiment, assuming that this might yield the predicted effects.

Our exploratory analysis of keypress B durations in the temporal condition revealed a repulsion effect, indicating that having planned the execution of a *long* keypress generally *shortens* actual keypresses that must occur before the planned response. The reason for such a repulsion effect might be that the system exaggerates the differences between the two action plans (or the two temporal codes within one two-response plan) to avoid confusing them.

## Experiment 2

Experiment 2 aimed to generate feature-binding effects by rendering the required action features for both action files less certain/predictable. For this purpose, the method of the first experiment was slightly adjusted, so to de-correlate Action and horizontal location. More specifically, participants were to plan Action A with either the *left* or *right* hand in an unpredictable manner. As in Experiment 1, Action B was then executed with the other hand, respectively, so that horizontal location always alternated. Accordingly, the horizontal feature for Action A was signaled no earlier than at Stimulus A onset, the horizontal feature for Action B could still be determined before onset of Stimulus B (i.e., also with onset of Stimulus A).

### Method

#### Participants

We collected data from *n* = 70 participants (33 females, 37 males, M_age_ = 26.8, range = [18,55]). Until this maximum sample size was reached, neither the RT, BF_10_ = 1.42, Median Cohen’s *d* = 0.23, 95% credibility interval (CI) = [0.03, 0.45], nor the ER data, BF_01_ = 5.73, Median Cohen’s *d* = 0.09, 95% credibility interval (CI) = [0.00, 0.29], had provided strong support for either hypothesis. Due to the more complex task, participants remained in the sample when they responded correctly in at least 60% of trials in both blocks, as preregistered. Still, of the overall 82 complete data sets, we excluded *n* = 9 participants for the vertical block, and *n* = 2 participants for the temporal block, as well as *n* = 1 participant who exceeded the error threshold in both blocks.

#### Procedure

The method in Experiment 2 was as in the previous experiment (see ***Figure 2***) except for the following points. Firstly, while in Experiment 1, letter stimuli occurred always for Action A and color stimuli for Action B, this could now also be the other way around. Put differently, one stimulus type still indicated a specific horizontal feature. Which stimulus type was used for the Stimuli A and B, that is, the order of stimulus types (first color, then letter or vice versa) and hence the order of the hands, was additionally varied. Secondly, the overall experiment was shortened from 80 repetitions per design cell to 64.

### Results

#### Balanced Integration Scores

The analysis of BIS for Action B showed worse performance in the vertical than the temporal condition, *F*(1,69) = 12.88, *p* = .001, \eta _p^2 = .16. Importantly, there were also general feature overlap costs, *F*(1,69) = 22.54, *p* < .001, \eta _p^2 = .25, which did not differ significantly between the vertical and the temporal feature types, *F*(1,69) = 2.81, *p* = .10, \eta _p^2 = .04.

#### Response Durations

The analysis of keypress B durations in the temporal condition yielded a significant main effect of Feature B, with longer durations for *long* than *short* keypresses, *F*(1,69) = 1698.39, *p* < .001, \eta _p^2 = .96, but, importantly, longer keypresses (122 ms vs. 120 ms for required *short* keypresses and 338 ms vs. 331 ms for required *long* keypresses) when a *short* than a *long* keypress A was planned, *F*(1,69) = 16.16, *p* < .001, \eta _p^2 = .19. In contrast to Experiment 1, this latter effect was not significantly stronger for any duration category, *F*(1,69) = 3.54, *p* = .06, \eta _p^2 = .05.

### Discussion

As predicted, the modest methodological adjustment changed the results drastically, generating substantial partial-overlap costs for both vertical and temporal features. Moreover, we replicated the repulsion effect observed in Experiment 1. These outcomes show that partial overlap costs cannot only be demonstrated for horizontal features, but for vertical and temporal features as well. Notably, however, this successful extension of the binding principle in action planning presupposed the reduction of feature certainty as compared to Experiment 1. We thus suggest that the absence of binding effects in Experiment 1 should not be attributed to the use of relatively simple keypressing responses—as compared to the more complex action sequences used by Stoet and Hommel (1999) and others, but to the lack of uncertainty regarding the horizontal action feature.

## Experiment 3

In Experiment 2, by design, the horizontal feature of Action B was already implied with onset of Stimulus A, meaning that participants had to extract only the second, vertical or temporal feature from Stimulus B. Experiment 3 extended on this by maximizing uncertainty of the horizontal feature of Action B as well, to see whether this would replicate or even enhance the effect found in Experiment 2. Therefore, now for both actions, which features would be required became apparent only after onset of the respective stimulus, thus with Stimulus B for Action B. To achieve this, we introduced hand repetition trials, which, however, would not become part of the analyses. They merely served the purpose of reducing certainty of Action B, which could now also rely on the same horizontal feature as Action A. Thus, although we used four possible trial types (full feature overlap, horizontal feature overlap, vertical/temporal feature overlap and no feature overlap), our analytical focus was on hand alternation trials to ensure comparability with the previous experiments, where repetitions were impossible by design.

### Method

#### Participants

After having a sample of *n* = 50 participants (21 females, 29 males, M_age_ = 27.6, range = [18,56]), the ER data provided strong support for the H_0_, that is, support against a significant difference in partial overlap costs between the vertical and the temporal conditions, BF_01_ = 15.03, Median Cohen’s *d* = 0.06, 95% credibility interval (CI) = [0.00, 0.20]. From the collected *n* = 53 complete data sets, we excluded three participants who showed more than 40% errors in the temporal block.

#### Procedure

The procedure was mostly the same as in Experiment 2. Stimulus types (i.e., letters or colors) were still assigned to one horizontal feature, and both stimulus types appeared equally often for Action A. Importantly, now also for Action B could both stimulus types be presented, irrespective of Stimulus A. This resulted in 50% hand alternation and 50% hand repetition trials. Each design cell thus contained 32 trials.

#### Data Analysis

The here reported analyses were identical to the previous experiment. However, ***Table 1*** reports descriptive results of the unanalyzed hand repetition conditions for the interested reader, in addition to the results of the full (2 × 2 × 2) model, including the within-subjects factor hand overlap presented online.

### Results

#### Balanced Integration Scores

The analysis of BIS for Action B in hand alternation trials showed worse performance in the vertical than the temporal condition, *F*(1,49) = 4.11, *p* = .048, \eta _p^2 = .08. There were also, again, feature overlap costs, *F*(1,49) = 15.15, *p* < .001, \eta _p^2 = .24, whereas the interaction between feature type and overlap was not significant, *F*(1,49) = 1.25, *p* = .27, \eta _p^2 = .03.

#### Response Durations

The analysis of keypress B durations in the temporal hand alternation trials yielded longer durations for *long* keypresses than for *short* ones, *F*(1,49) = 1314.09, *p* < .001, \eta _p^2 = .96, but, again, also longer keypresses (119 ms vs. 115 ms for required *short* keypresses, and 346 ms vs. 341 ms for required *long* keypresses) when a *short* than a *long* keypress A was planned, *F*(1,49) = 5.63, *p* = .022, \eta _p^2 = .10. The latter effect was again not significantly stronger for either duration category, *F*(1,49) = 0.08, *p* = .78, \eta _p^2 < .01.

### Discussion

Experiment 3 replicated the finding that vertical and temporal features can be bound and retrieved in action planning, as well as the duration repulsion effect. Since we knew already that reducing certainty of Action A and Action B features (as was done from Experiment 1 to 2), can increase partial overlap costs, we wondered whether reducing Action B certainty even more, to the minimum, would change the results. The data do not necessarily support this idea, as the partial overlap costs in BIS (averaged over feature type conditions) did not differ significantly between Experiments 2 (M = 0.57, SD = 1.00) and 3 (M = 0.69, SD = 1.26), *t*(118) = 0.59, *p* = .55 (two-sided), *d* = 0.11. A corresponding Bayesian *t*-test yielded moderate evidence for the null hypothesis, BF_10_ = 0.23, error % < 0.01% (using default priors). This observation suggests that, when planning an Action A, and first executing another Action B, binding and retrieval of the codes of (here: horizontal) Action A features depends on the (un)certainty of these exact features and less on the (un)certainty of the (horizontal) Action B features that could possibly suffer from incorrect action file A retrieval.

## Experiment 4

After having demonstrated binding and retrieval of vertical and temporal features in our paradigm, provided sufficient uncertainty of the to-be-combined features, the question remained why we had not found the expected effect in the spatial condition in Experiment 1 already, despite it being a conceptual replication of Stoet and Hommel (1999). To shed light on this issue, we had a closer look at the subtle differences between our first experiment and Experiment 2 of Stoet and Hommel (1999).

Firstly, although the two experiments used the same (i.e., vertical and horizontal) features, Stoet and Hommel (1999) employed hand and feet as *top* and *bottom* effectors, while our present vertical dimension referred to the location of fingers. Arguably, agents might be more used to discriminate between their hands and their feet in terms of vertical location (e.g., when deciding which effector to use to touch or kick objects in high or low locations) than to discriminate their fingers in these terms, which might have affected the likelihood or proficiency in vertical coding.

Secondly, Stoet and Hommel (1999) used a more complex action sequence for Action A and releases instead of presses for Action B. Their reason was that simpler actions might not be planned upon presentation of the corresponding stimulus; rather, participants might tend to memorize the stimulus and start planning no earlier than right before execution. If so, the features of Action A would not yet be bound when planning Action B, so that no binding effects would be expected. The present Experiments 2 and 3 clearly show that binding effects can be demonstrated with keypresses as well, so that we do not consider this account to provide a likely explanation of the lack of binding effect in Experiment 1.

Thirdly, in the Stoet and Hommel (1999) study, certain features were vertical (*top*/*bottom*) and uncertain features horizontal (*left*/*right*). In Experiment 1, we had participants plan Action A with, for example, the *left* hand and execute Action B with the *right* hand, while Stoet and Hommel (1999) had participants plan a *manual* response and execute a *pedal* response first. In their experiment, thus, the horizontal features *left* and *right* remained uncertain until the eventually required action was signaled, while the features *hand* and *foot* were assigned to Actions A and B in advance. By contrast, in our Experiment 1, the horizontal features *left* and *right* were known in advance while the vertical features *top* and *bottom* remained uncertain until the eventually required action was signaled. Horizontal features seem to decay more quickly than vertical features (Vallesi et al. 2005; Wiegand & Wascher 2005). If they vary from trial to trial, they might thus require stronger binding with other features to create a temporarily stable action plan. Thereby, the potential of horizontal features to retrieve other features accidentally when becoming part of still another action – the likely cause of partial overlap costs (see Frings et al. 2020) – might increase as well.

Based on these considerations, we decided to test the idea that partial overlap costs are particularly likely if horizontal features vary unpredictably. We thus conducted Experiment 4, in which the vertical condition now more closely resembled the experiment by Stoet and Hommel (1999), as Action A predictably required, for example, a press of one of the two *top* keys (corresponding to the *hands* in the original study), and Action B a press of one of the two *bottom* keys (corresponding to the *feet*). Similar to the original experiment, stimuli then indicated whether the *left* or *right* hand would have to be used. While the focus was on examining the effect of switching the roles of the vertical and horizontal features, we added the temporal condition mainly for consistency with the previous experiments and for exploratory purposes (i.e., participants always planned, for instance, a *short* keypress for Action A and executed a *long* keypress for Action B). Taken together, horizontal features could overlap while Actions A and B always differed with respect to the vertical (temporal) feature.

### Method

#### Participants

After testing *n* = 40 participants (20 females, 20 males, M_age_ = 25.4, range = [18,40]), the ER data provided strong support for the H_1_, BF_10_ = 24.01, Median Cohen’s *d* = 0.47, 95% credibility interval (CI) = [0.15, 0.80]. From the collected *n* = 52 complete data sets, we had excluded participants who showed more than 40% errors in the temporal block (*n* = 6), in the vertical block (*n* = 4), or in both blocks (*n* = 2).

#### Apparatus and Stimuli

Participants positioned their hands on the keyboard as in all previous experiments, and the procedure was as in Experiment 1. However, instead of mapping stimulus types to the two horizontal features, as in Experiment 1, we now mapped stimulus types to the two vertical features in the spatial block (i.e., *top* responses to letter stimuli and *bottom* responses to color stimuli or vice versa) or the two *temporal* features in the temporal block (i.e., *short* responses to letter stimuli and *long* responses to color stimuli or vice versa). This means that Actions A and B always alternated in terms of either vertical location (e.g., first *top*, then *bottom*) or temporal duration (e.g., first *short*, then *long*). The horizontal features were signaled by the particular stimuli, so that one stimulus (e.g., ‘X’ and red) signaled the *left* key and the other (e.g., ‘O’ and green) the *right* key.

### Results

Overall performance, measured as BIS, did not differ between the vertical and the temporal condition, *F*(1,39) = 0.20, *p* = .654, \eta _p^2 < .01. There was also no main effect of feature overlap, *F*(1,39) < 0.01, *p* = .97, \eta _p^2 < .01. However, the interaction between feature type and overlap was significant, *F*(1,39) = 10.91, *p* = .002, \eta _p^2 = .22, due to overlap costs failing to reach significance in the vertical condition, *t*(39) = 1.98, *p* = .054, *d_z_* = 0.31, and overlap benefits reaching significance in the temporal condition, *t*(39) = 2.46, *p* = .018, *d_z_* = 0.39.

### Discussion

When switching the vertical and horizontal feature types to align with the original study by Stoet and Hommel (1999), we found signs of partial overlap costs in the spatial condition, even though they were not significant. One might infer from this that uncertain, possibly overlapping, horizontal features (as was the case in almost every previous study) provoke binding and retrieval irrespective of the certainty of the other features, whereas this does not apply to vertical or temporal features (Experiment 1). Yet, when also considering the temporal feature condition of this fourth experiment, the case turns out to be even more complex. With certain temporal features and uncertain horizontal features, we found major overlap benefits, whereas we found overlap costs when temporal and horizontal features varied from trial to trial (Experiments 2 & 3). Sticking with the line of argument that feature variability promotes stronger binding of these features would suggest that temporal features bind with other features only if they both are highly variable (Experiments 2 & 3). Altogether this set of experiments suggests that feature uncertainty fosters the observation of feature binding, while at the same time different types of features are differently affected by such uncertainty.

## General Discussion

### Binding of Temporal Features

Our main research question was whether partial overlap with respect to other (non)spatial action features than horizontal (*left* or *right*) can create partial overlap costs that are diagnostic for integration of these features in and retrieval from action plans. To investigate this, we created partial feature overlap with vertical (*top* or *bottom*) or temporal (*short* or *long*) action features, rather than with horizontal features (*left* or *right*) as in previous research. We found that, in principle, these feature types can give rise to partial overlap costs, similar to horizontal features. Yet there were also some differences in binding vertical and temporal features which we will consider below.

### Feature Uncertainty

The lack of partial overlap costs in Experiment 1, in contrast to Experiments 2 and 3, suggests that feature uncertainty generally enhances feature binding in (and probably accidental retrieval from) action plans (see ***Figure 2***). For example, the horizontal feature of Action A could be determined not before onset of Stimulus A in Experiments 2 and 3. Thus, participants had to extract all required Action A features from Stimulus A, and feature overlap with the vertical/temporal feature in Action B then evoked partial overlap costs. Further, the fact that the results in Experiments 2 and 3 do not seem to differ substantially, suggests that the crucial factor is in fact the certainty of Action A features prior to Stimulus A onset, and not the certainty of Action B features prior to Stimulus B onset. Put differently, whether the horizontal feature of Action B was signaled by the onset of Stimulus A, which was the case in Experiment 2 (as the horizontal features never overlapped), or by onset of Stimulus B, as in Experiment 3, seemed to be of less importance.

The observation that partial feature overlap costs are more prominent in cases where there is high uncertainty about momentarily needed features is, first of all, an important methodical issue. The general recommendation from this study for those who want to explore feature binding and retrieval in action planning is probably to render all momentarily needed features of an action as uncertain or unpredictable as possible. A somewhat different strategy was used by Stoet and Hommel (1999), who used more complex Actions A with open (uncertain) parameters: participants were uncertain with regard to (a) which hand should move and which home key was to be released as a first step; (b) whether the hand should then move up or down in the second step; (c) whether it should move up or down in the third step (even though this was negatively correlated with the direction of the second step); and whether it should move up or down to go back to the home key. Given substantial evidence that actions with so many open parameters strongly promote online action planning (e.g., Rosenbaum 1985, 1987), using more complex actions are likely to generate binding effects even if the response set is small. Studies with less complex actions, however, should consider introducing more uncertainty by reducing the number of fixed parameters and/or by increasing the response set (i.e., the combinations of features defining the responses). That uncertainty about to-be-planned features of actions is important at all fits with the original idea of theorists to introduce binding as a mechanism that deals with the problem that it is often the combination of features that matter for performance (Kahneman, Treisman & Gibbs 1992). If every feature of a certain type can go together with every feature of another type at essentially any point in time, feature codes are likely to compete with each other, and action files are needed to organize this competition.

The more the need of a certain feature for a certain occasion becomes predictable, the lower the feature competition for one action file „slot“, and the more likely it seems that this certain feature enters a more long-term memory structure that encompasses both actions, A and B. In other words, people may plan a sequence of two actions, with two feature slots being permanently filled (e.g., ‘*short*-then-*long’*). Indeed, research on action planning has shown that the time to plan a particular action increases with the number of open parameters as long as all combinations of the possible features are equally likely, but this dependency of planning time on the number of feature dimensions tends to disappear if feature values become contingent on each other (i.e., if particular feature combinations are more likely than others, Lépine, Glencross & Requin, 1989). The same may hold for feature binding: reducing uncertainty regarding particular feature values might promote the integration of action plans for Actions A and B, so that binding may still occur but in more complex ways that no longer need to produce partial-overlap costs that we took as indicator of binding. Whatever the specific reasons for the impact of feature uncertainty are, they obviously differ between feature types, at least in designs with simple actions.

### Limitations and Future Investigation of Binding and Retrieval

The present study comes with limitations. First, we relied on a combined measure of RTs and ERs, as influences of feature overlap did not selectively show up in one of these measures. While we had noted this peculiarity already during the course of experimentation and preregistered this analytical approach for the remaining experiments, it is certainly desirable to find ways to channel experimental effects into one dependent measure. We speculate that the conduction as online experiments came with lesser focus on accuracy than one would expect from participants that are tightly monitored by an experimenter. Second, as almost every study, we relied on a comparison between partial feature overlap and no feature overlap. Theoretically, full feature overlap should come with performance in the range of no feature overlap (Frings et al. 2020)—but there are obvious mechanical obstacles to realize this condition in many cases (e.g., if it implies using the same effector twice). For a more complete picture, future action planning studies may find ways to realize the full design, including complete feature overlap (see Mocke et al. 2020, for such an approach).

## Conclusion

The issue of what determines feature binding during action planning and retrieval from unexecuted action plans turned out to be much more complex than initially expected. We inferred from the data that other (non-)spatial features than horizontal ones can be bound and retrieved, and additionally identified another factor that seems to hinder binding/retrieval, namely, feature certainty. Many of our findings gave rise to even more questions, but especially due to the complexity of this issue is this series of experiments an important first step towards understanding under which conditions feature codes of planned actions are bound and retrieved.

## Data Accessibility Statement

Preregistrations, raw data, and analyses are available on the the *Open Science Framework* (*https://doi.org/10.17605/OSF.IO/7QYH4*).
